# Chronic allergic asthma alters m^6^A epitranscriptomic tagging of mRNAs and lncRNAs in the lung

**DOI:** 10.1042/BSR20221395

**Published:** 2022-12-12

**Authors:** Xiuqin Ni, Xing Li, Bing Hu, Li Wang

**Affiliations:** 1 Department of basic medicine, School of Health Sciences, Jiangsu Food & Pharmaceutical Science College, Huai’an 223003, China; 2 Department of Anatomy, Harbin Medical University-Daqing, Daqing 163319, China; 3 Department of Neurology, Hongze District People’s Hospital, Huai’an 223001, Jiangsu Province, China

**Keywords:** allergic asthma, epitranscriptome, IL17RB, lung, N6-methyladenosine

## Abstract

To evaluate the role of m^6^A methylation of mRNAs and long non-coding RNAs (lncRNAs) in chronic allergic asthma. Transcriptome-wide N6-methyladenosine (m^6^A) changes in BALB/c mice were profiled using immunoprecipitated methylated RNAs with microarrays in lung with chronic allergic asthma. Gene ontology (GO) and KEGG analyses were conducted. Target genes were verified by methylated RNA immunoprecipitation and real-time polymerase chain reaction (PCR). Specifically, the mRNA levels of m^6^A writers (METTL3, METTL14, and WTAP), and readers and erasers (FTO and ALKBH5) were estimated by real-time PCR analysis, using the SYBR-green method. IL17RB mRNA was also evaluated by PCR. Hematoxylin and eosin (H&E) staining showed that the airway and lung tissues in mice in the asthma group had extensive infiltration of inflammatory cells around the bronchioles, blood vessels, and alveoli. The lungs of those allergic asthma mice showed altered m^6^A epitranscriptome, whereby 1369 mRNAs and 176 lncRNAs were hypermethylated, and 197 mRNAs and 30 lncRNAs were hypomethylated (>1.5-fold vs control). Also, compared with the control group, IL17RB mRNA in lung of the asthmatic group was significantly hypermethylated (*P*<0.01). In the asthma group, the mRNA and the protein level of METTL14 (the key methyltransferase) and ALKBH5 (the major demethyltransferase) were significantly decreased compared with the control group (*P*<0.01). Chronic allergic asthma alters the lung m^6^A epitranscriptome, suggesting functional implications in the pathophysiology of refractory asthma. Data support methylated IL17RB mRNA possibly becoming a new therapeutic target for chronic allergic asthma.

## Introduction

Allergic asthma is characterized by a series of immune regulatory events and molecular events. This includes helper T cell 1 (Th1)/helper T cell 2 (Th2) imbalance, helper T cell 17 (Th17)/regulatory T cell (Treg) imbalance, inflammation, phenotypic transformation, and more [1,2]. In addition, diverse post-transcriptional regulators of RNAs involving various classes of noncoding RNAs, RNA-binding proteins (RBPs), and epitranscriptomic modifications can finely regulate immunologic and pathophysiological changes associated with allergic asthma [3,4]. Long non-coding RNA (lncRNA) promotes the proliferation and migration of airway smooth muscle cells, airway inflammation, and Th2 inflammatory response in asthma [4–7]. Moreover, exposure to environmental toxins is closely related to the differentially N6-methyladenosine (m^6^A)-methylated modification in mRNAs, which is part of the immunomodulatory process [7].

Recently, many studies have discovered that RNAs could undergo more than 100 types of post-transcriptional modifications, which are defined as epitranscriptomic changes [8,9]. Among these modifications, m^6^A RNA methylation is the most prevalent [10], and can play a biological role in two ways. First, it can change the structure of local RNA, allowing it to enhance RNA–protein interactions. Second, the recruited specific protein binds to the m^6^A-modified site.

Studies have confirmed that m^6^A modification plays a role in the transcription, translation, and degradation of mRNA [11,12]. The m^6^A modification is mainly located in RRACH motifs, which refers to R as a purine base, A as adenine, C as cytosine, and H as a non-guanine base. In mammals, modification occurs by a methyltransferase complex (dedicated writers) consisting of METTL3, METTL14, WTAP, and other components. This modification can be reversed by the demethylases (dedicated erasers) FTO and ALKBH5 [13–15]. Studies have reported that m^6^A methylation modification may affect an array of life processes, such as sperm development, development of immunity, UV-induced DNA damage response, tumorigenesis or metastasis, stem cell renewal, fat differentiation, biological rhythms, cell development and differentiation, and cell division [16–18]. Thus, studies clearly indicate that changes to core genes are sensitive to the function of m^6^A modulation, which can cause epigenetic changes.

Studies have also shown that the increased m^6^A methylation of transcripts is related to inflammation and immune regulation. Other findings indicate that YTHDF2, an m^6^A reader can regulate the LPS-induced inflammatory response [19]. Differential levels of m^6^A-methylated mRNAs after stroke can regulate biological processes including inflammation, apoptosis, and transcriptional regulation [9]. Also, m^6^A methylation of transcripts has an effect on the adaptive immune response by acting on the stability of innate immune molecules [20]. Moreover, a paper by Han et al. proposed that m^6^A methylation of mRNAs in dendritic cells can control antitumor immunity [21]. There are additional findings, namely that m^6^A modification can promote pulmonary fibrosis [22], and may be involved in immunoregulation [7]. Currently, however, our knowledge of the role of m^6^A modifications in allergic asthma remains limited. As a result, we aimed to explore m^6^A modification of mRNAs and lncRNAs in lung tissues of mice with allergic asthma, which might reveal a new pathophysiological mechanism, and possibly a new therapeutic target for allergic asthma.

## Materials and methods

### Ovalbumin-sensitized and challenged for allergic asthma model

Twelve BALB/c mice were provided by the Laboratory Animal Research Center in Beijing, China. The mice (6- to 8-week-old, female) were housed in pathogen-free conditions, and were randomized into two groups: the control group and the asthma group. Ovalbumin (OVA)-sensitized and challenge protocols for the chronic model of asthma were carried out according to the methods described by Daubeuf et al. [23], Vieira et al. [24], and Penton et al. [25] with a minor modification. Briefly, the mice in the asthma group were sensitized with 0.01% OVA by intraperitoneal injection on Days 0 and 7, and were subsequently challenged with 2.5% OVA aerosols three times weekly for 7 weeks. The mice in the control group were injected and challenged with PBS. The animals were executed by cervical dislocation 24 h after the last OVA or PBS exposure. All animal experiments and manipulation procedures were done in Harbin Medical University-Daqing and approved by the Ethics Committee for Animal Use and Care at the Institute of Education of Harbin Medical University-Daqing, China.

### Hematoxylin and eosin staining

The left lung lobe was fixed with 4% paraformaldehyde, then routinely embedded in paraffin and sectioned (5 µm) for hematoxylin and eosin (H&E) staining. The slides were observed by microscope.

### m^6^A immunoprecipitation

Total RNA (3 μg) and m^6^A spike-in control mixture were added to IP buffer (50 mM Tris-HCl, pH 7.4, 150 mM NaCl, 0.1% NP40, 40 U/μL RNase inhibitor) containing 2 μg anti-m^6^A rabbit polyclonal antibody (Synaptic Systems). The reaction was incubated with head-over-tail rotation at 4°C for 2 h. Dynabeads™ M-280 sheep antirabbit IgG beads per sample were blocked with freshly prepared 0.5% BSA at 4°C for 2 h, washed three times with IP buffer, and then resuspended in the total RNA-antibody mixture prepared above. The RNA binding to the m^6^A-antibody beads was carried out with head-over-tail rotation at 4°C for 2 h. The beads were then washed three times with IP buffer, and twice with wash buffer (50 mM Tris-HCl, pH 7.4, 50 mM NaCl, 0.1% NP40, 40 U/μL RNase Inhibitor). The enriched RNA was eluted with elution buffer (10 mM Tris-HCl, pH 7.4, 1 mM EDTA, 0.05% SDS, 40 U Proteinase K) at 50°C for 1 h. The RNA was extracted by acid phenol–chloroform and ethanol precipitation.

### Labeling and microarray hybridization

The immunoprecipitated RNA samples from the control and asthma groups (*n*=3 each) were labeled with Cy5 fluorescent dye using a Super RNA Labeling Kit (Arraystar), and purified using an RNeasy Mini Kit. Cy5-labeled cRNAs were fragmented and hybridized to a mouse m^6^A-mRNA and lncRNA epitranscriptomic microarray (8×60K, Arraystar) containing 48161 mRNA and 8393 lncRNA degenerate probes. The hybridized arrays were washed, fixed, and scanned using an Agilent Scanner G2505C.

### Microarray data analysis

Agilent Feature Extraction software (version 11.0.1.1) was used to analyze acquired array images. Raw intensities of immunoprecipitated RNAs were normalized with an average log2-scaled spike-in RNA control. Differentially m^6^A-methylated RNAs between the control and asthma groups were identified by filtering with the fold-change and statistical significance (*P*-value) thresholds. A cutoff of 1.5-fold (*P*<0.05) was used to identify the differentially m^6^A-methylated RNAs in asthma samples as compared with control. Hierarchical clustering was performed using R software. Gene ontology (GO) analysis was performed using the topGO package in the R environment for statistical computing and graphics. Pathway analysis was calculated by the Fisher’s exact test.

### Real-time polymerase chain reaction

Total RNA (1.5 µg) was reverse transcribed to cDNA with a High Capacity RNA to cDNA Kit (Applied Biosystems). The mRNA levels of m^6^A writers (METTL3, METTL14, and WTAP), readers and erasers (FTO and ALKBH5) were estimated by real-time polymerase chain reaction (PCR) analysis with the SYBR-green method. IL17RB mRNA was also evaluated by PCR. For methylated RNA immunoprecipitation-quantitative PCR (MeRIP-qRT-PCR) analysis of differentially methylated RNAs, 3 µL immunoprecipitated RNA, or 1 µL input RNA was converted into cDNA and amplified with gene-specific primers for mRNAs (CCL11, TNF, CCL2, CXCL1, CCL8, IL17RB, and IL5). The primers used in the present study are presented in Tables 1 and 2.

**Table 1:** Sequences of primers used for qRT-PCR analysis of mRNA levels

Name	Sequence	Product size (bp)
CCL11	F:5′ TGCTTGATTCCTTCTCTTTCCT 3′	228
	R:5′ TTACTCCTAACTCGTCCCATTGT 3′	
TNF	F:5′ TCTACTGAACTTCGGGGTGAT 3′	83
	R:5′ TCTGGGCCATAGAACTGATGA 3′	
CCL2	F:5′ CCTGCTGTTCACAGTTGC 3′	110
	R:5′ TCATTGGGATCATCTTGC 3′	
CXCL1	F:5′ ACCCAAACCGAAGTCATAGCC 3′	169
	R:5′ AGAAGCCAGCGTTCACCAGA 3′	
CCL8	F:5′ GTGCTGAAAAGCTACGAGAGAAT 3′	223
	R:5′ CCTGGAGAAGATTAGGGGAGA 3′	
IL17RB	F:5′ AAGGAAGGAGCACGAAGACG 3′	109
	R:5′ CGACAGACGGTGTGATGGAA 3′	
IL5	F:5′ CTCCTCAACTCCCTGCTACTCTC 3′	194
	R:5′ TGTGGCTGGCTCTCATTCAC 3′	

**Table 2:** Sequences of primers used for MeRIP-qRT-PCR analysis of m^6^A methylation levels

Name	Sequence	Product size (bp)
GAPDH	F:5′CACTGAGCAAGAGAGGCCCTAT3′	144
	R:5′GCAGCGAACTTTATTGATGGTATT3′	
METTL3	F:5′ CGGACACGTGGAGCTCTATC3′	153
	R:5′ GCACGGGACTATCACTACGG3′	
METTL14	F:5′ ACCTCCTCCCAAGTCCAAGT3′	123
	R:5′ CCACCTCTCTCTCCTCGGAA3′	
WTAP	F:5′ GTGTGGCCCAACTGAGATCA3′	206
	R:5′ CACCACAGCTTTCTCGAGGT3′	
ALKBH5	F:5′ GGGACAGAGGAGCTCAGGTA3′	193
	R:5′ TGGAGAGCTGCTGACAGTTG3′	
FTO	F:5′ AGCCCCTGGGTCTCCATAAT 3′	174
	R:5′ AGCACTCAACGCTACTGCTT 3′	
IL17RB	F:5'GAGAGTGAAGGTGCGGTGGTT3′	131
	R:5′CGTCTGTTGTCATCTGGAGGG3′	

### Western blotting

Protein were extracted from lung tissues with RIPA lysis buffer obtained from Beyotime in China. Protein quantification was done with Enhanced BCA Protein Assay Kit (Beyotime). Equal amounts of lysate proteins were separated by using SDS-PAGE and transferred to nitrocellulose membranes. After blocking in 5% nonfat milk, the membranes were incubated with the primary antibodies against METTL3 (Cat. No. ab195352, 1:1000 dilution), METTL14 (Cat. No. A8530, 1:500 dilution), WTAP (Cat. No. A8530, 1:500 dilution), FTO (Cat. No. ab280081, 1:1000 dilution), ALKBH5 (Cat. No. ab195377, 1:1000 dilution), β-actin (1:1000; Santa Cruz Biotechnology) overnight at 4°C, followed by a 1 h incuabation with secondary HRP antibodies at a room temperature. All protein blots were visualized using enhanced chemiluminescence reagents (GE Healthcare Life Sciences) and exposure to X-ray.

### Statistical analysis

GraphPad Prism 5.0 software was used in the analysis.

## Results

### m^6^A modification profiles in lungs of allergic asthma mice

**Figure 1:**
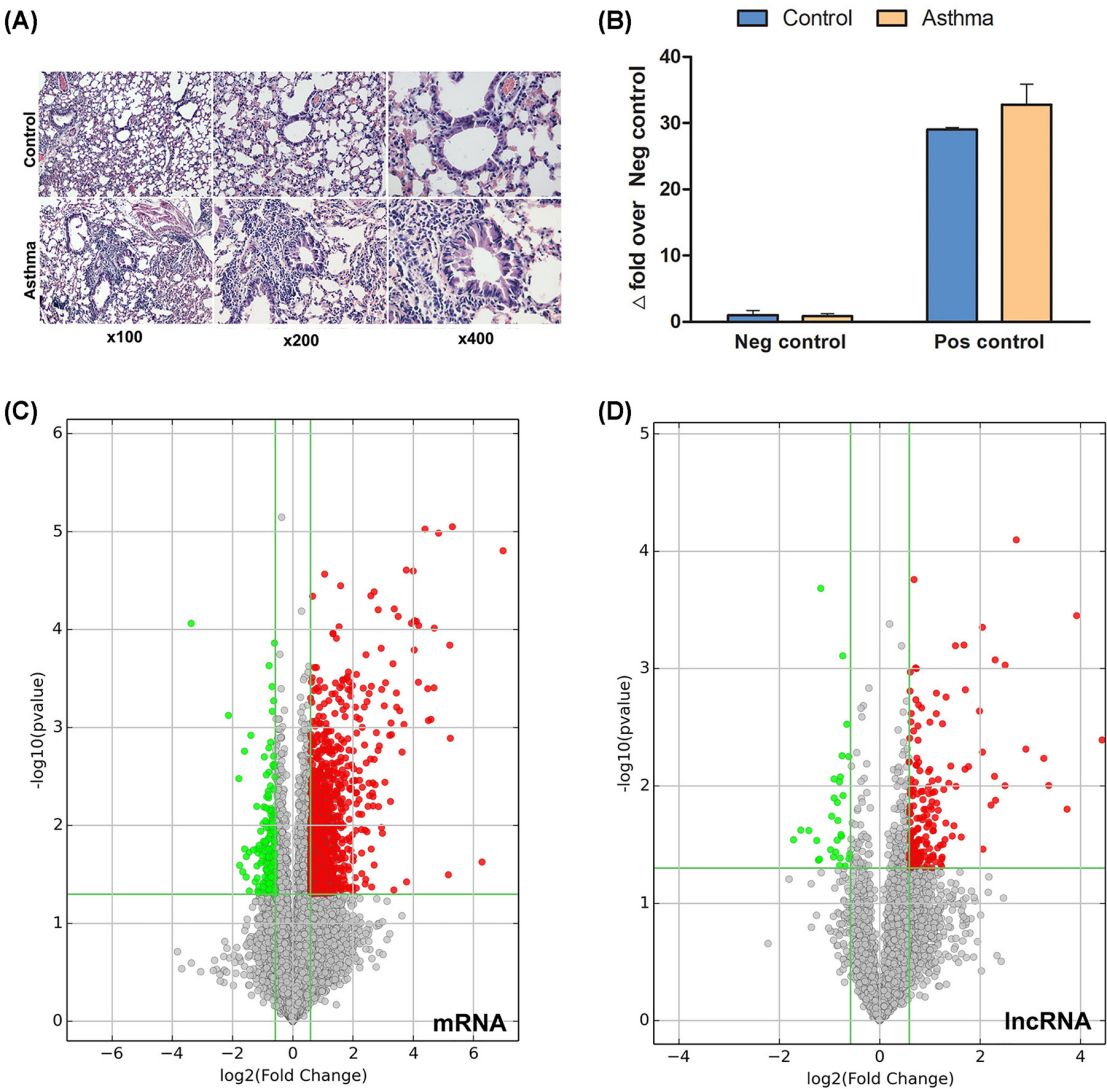
Chronic allergic asthma significantly alters the lung m^6^A epitranscriptome (**A**) Pathological changes in lung of chronic allergic asthma model. Obvious bronchiolar stenosis can be seen in the asthmatic model group, and the bronchiolar and lung tissues show obvious inflammation and inflammatory cell infiltration (Original magnification: left column ×100, middle column ×200, right column ×400). (**B**) Assessment of MeRIP quality. The identical methylated RNA immunoprecipitation enrichment of positive and negative spike-in controls was observed via normalizing % (MeRIP/Input) of the positive with negative controls for the asthma and control groups. Data are mean ± SEM (*n*=3). *P<0.05 compared with the control by the Mann–Whitney U test. Volcano plots for (**C**) mRNAs and (**D**) lncRNAs. m^6^A hypermethylated (red dots) and hypomethylated (blue dots) in the asthma group over the control group (*n*=3). The green threshold lines are set at 1.5-fold (*P*<0.05) between the asthma and control. Abbreviations: MeRIP, methylated RNA immunoprecipitation; Neg, negative; Pos, positive.

H&E staining showed the pathologic changes of chronic allergic asthma. Specifically, it detected that the airway and lung tissues in mice of the control group had normal smooth bronchial and alveolar structures, and no inflammatory cell infiltration. In contrast, the airway and lung tissues in mice of the asthma group showed extensive infiltration of inflammatory cells around the bronchioles, blood vessels, and alveoli. The airway mucosa had obvious edema, with bronchiolar wall thickening causing luminal stenosis. This finding indicated the successful establishment of the asthmatic model (Figure 1A).

The immunoprecipitated m^6^A-methylated RNAs isolated from the lung of mice were profiled in both the asthma and control groups. By assessment of MeRIP quality, the identical methylated RNA immunoprecipitation enrichment of positive and negative spike-in controls was observed in both groups, indicating the rigor of the technique (Figure 1B). Using a microarray with probes for 56554 RNAs (48161 mRNA and 8393 lncRNA), the methylated RNAs microarray profiling showed that 1772 transcripts (1566 mRNAs and 206 lncRNAs) were differentially m^6^A methylated between the asthma and control groups (fold-change >1.5, *P*<0.05; *n*=3/group). The data showed that 87.2% of the transcripts (1369 mRNAs and 176 lncRNAs) were significantly hypermethylated, and only 12.8% of the transcripts (197 mRNAs and 30 lncRNAs) were significantly hypomethylated in the asthma group compared with the control group (Figure 1C,D). The top 20 altered m^6^A quantities are listed in Tables 3 and 4.

**Table 3:** The top 20 differently expressed m^6^A-methylated mRNA with allergic asthma

Gene name	Fold-change	Regulation	Locus	Strand	*P*-value
Rnase2a	126.3905758	Up	chr14:51255262-51256112:-	-	1.57095 × 10^-5^
Clca1	77.88635836	Up	chr3:145003817-145032776:-	-	0.023637391
Ptgdr	39.34120279	Up	chr14:44851235-44859375:-	-	8.93974 × 10^-6^
Cd209e	37.49292175	Up	chr8:3847965-3854309:-	-	0.001287945
RP23-101F14.3	37.08413836	Up	chr2:122637915-122641085:+	+	0.000144605
Slc22a29	35.85665903	Up	chr19:8160165-8218899:-	-	0.031886612
Arg1	28.62280631	Up	chr10:24915221-24927484:-	-	1.03858 × 10^-5^
Slc26a4	25.93901282	Up	chr12:31519827-31559969:-	-	9.68448 × 10^-5^
Chil4	25.72891127	Up	chr3:106201490-106219507:-	-	0.000393617
Gcnt3	24.00909968	Up	chr9:70031496-70038088:-	-	0.000826165
Fabp1	10.38350125	Down	chr6:71199827-71205023:+	+	8.66167 × 10^-5^
Fpr1	4.399144107	Down	chr17:17876471-17883940:-	-	0.000753326
Apold1	3.458800416	Down	chr6:134981718-134986836:+	+	0.003328392
Zc3h18	3.396808743	Down	chr8:122411325-122416248:+	+	0.025575942
Tbc1d4	3.16324158	Down	chr14:101462974-101609191:-	-	0.029726563
Hist1h3d	3.040476346	Down	chr13:23575763-23577011:+	+	0.001746882
Zfp287	3.029790124	Down	chr11:62721580-62728671:-	-	0.017421449
Dnajc28	2.924521477	Down	chr16:91614257-91618999:-	-	0.033642474
Tmem252	2.915554082	Down	chr19:24674008-24679661:+	+	0.020814451
Gemin8	2.736589715	Down	chrX:166178402-166180792:+	+	0.018761716

**Table 4:** The top 20 differently expressed m^6^A-methylated lncRNA with allergic asthma

Gene name	Fold change	Regulation	Locus	Strand	*P*-value
RP23-71C23.1	21.57256849	Up	chr12:106131978-106145400:-	-	0.004054933
Fgf18	15.18458121	Up	chr11:33116978-33147400:-	-	0.000353985
Ntmt1	13.30815266	Up	chr2:30807994-30816642:+	+	0.015777808
4930542D17Rik	10.32573787	Up	chr16:50590503-50654166:+	+	0.009903069
RP24-118B12.1	9.651769053	Up	chr5:33293614-33311150:-	-	0.005818338
RP23-309A11.2	7.531065684	Up	chr12:103895897-103898784:+	+	0.004853991
RP24-226D9.3	6.601355773	Up	chr6:89269511-89285725:-	-	8.00499 × 10^-5^
Saa3	5.657239913	Up	chr7:46711999-46713749:-	-	0.000933919
Nfyc	5.646882555	Up	chr4:120757954-120762175:-	-	0.009958198
RP23-272P19.8	4.952742877	Up	chr16:19212660-19236776:-	-	0.013246835
RP24-182L3.1	3.282208743	Down	chr16:13463636-13468755:-	-	0.028717785
Brox	2.969035563	Down	chr1:183290843-183297256:-	-	0.02378089
RP24-95P4.2	2.657611174	Down	chr1:57200645-57214993:-	-	0.023973675
RP23-334L22.1	2.382843798	Down	chr12:117602039-117603763:+	+	0.029132554
RP23-157H17.2	2.327912227	Down	chr9:74847983-74852873:+	+	0.042861024
RP23-132N23.1	2.292898301	Down	chr2:25141797-25145396:-	-	0.042042913
RP23-436D9.1	2.254236114	Down	chr11:18467479-18493255:-	-	0.000207255
RP24-496C22.4	1.967449237	Down	chr5:44700876-44706890:-	-	0.034964827
Gm17276	1.936717635	Down	chr17:32113429-32141839:+	+	0.018099091
RP23-153M14.4	1.888809851	Down	chr4:127035681-127049398:+	+	0.040218634

### GO analysis and pathway analysis of differentially methylated mRNA

**Figure 2:**
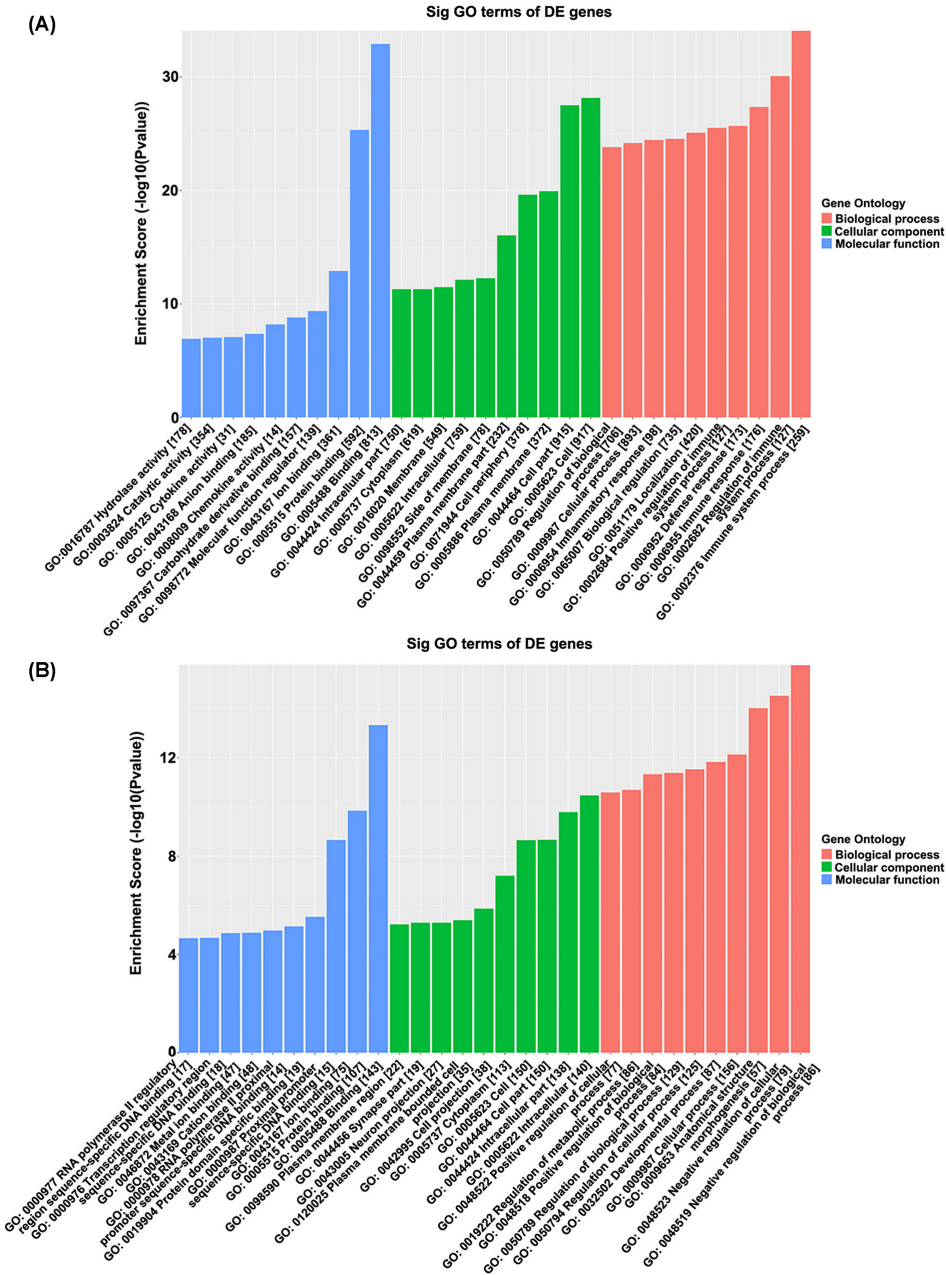
Altered m^6^A-modified transcripts contribute to the pathophysiology of allergic asthma (**A**) GO enrichment of up-regulation m^6^A transcripts. (**B**) GO enrichment of down-regulation m^6^A transcripts. DE genes refer to differentially expressed genes.

**Figure 3:**
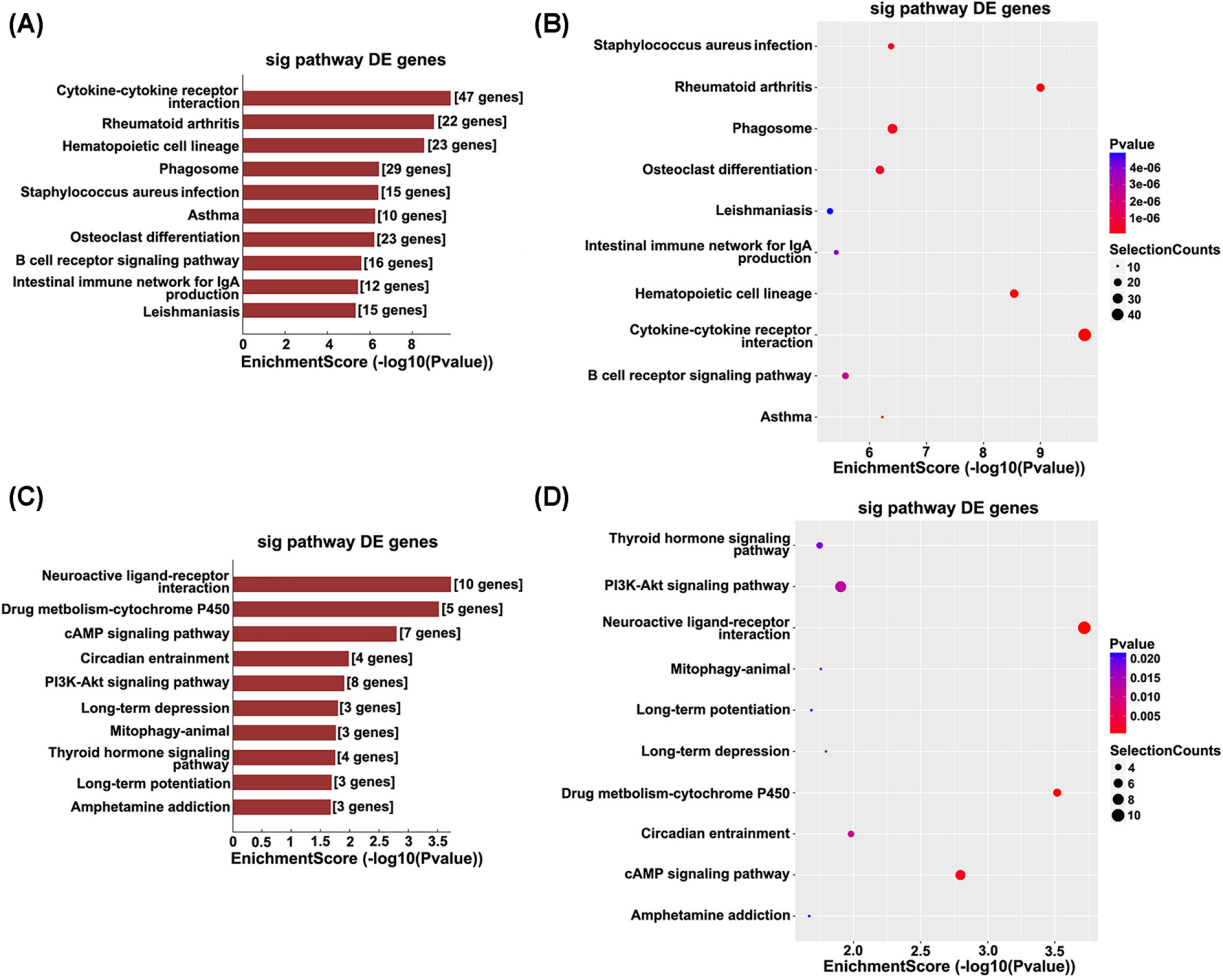
The top modified transcripts of altered m^6^A relating to the pathophysiology of allergic asthma The top ten significantly enriched pathways of up-regulation m^6^A transcripts by KEGG analysis (**A,B**). The top ten significantly enriched pathways of down-regulation m^6^A transcripts (**C,D**). DE genes refer to differentially expressed genes.

The physiological and pathological significance of m^6^A modification in allergic asthma were explored by GO analysis and KEGG pathway analysis. GO analysis illustrated that the up-regulated genes in allergic asthma were significantly associated with the immune system process and its regulation (ontology: biological process), cell and cell part (ontology: cellular component), and binding and protein binding (ontology: molecular function) (Figure 2A). The down-regulated genes were significantly associated with negative regulation of biological and cellular processes (ontology: biological process), intracellular and intracellular part (ontology: cellular component), and binding and protein binding (ontology: molecular function) (Figure 2B). Pathway analysis showed that up-regulated genes in the allergic asthma were significantly associated with cytokine–cytokine receptor interaction and rheumatoid arthritis (Figure 3A,B). The down-regulated genes were significantly associated with neuroactive ligand–receptor interaction and drug metabolism-cytochrome P450 (Figure 3C,D). All GO and pathway significant *P*-values were less 0.05.

### METTL14 and ALKBH5 were down-regulated in lung of mice with allergic asthma

**Figure 4:**
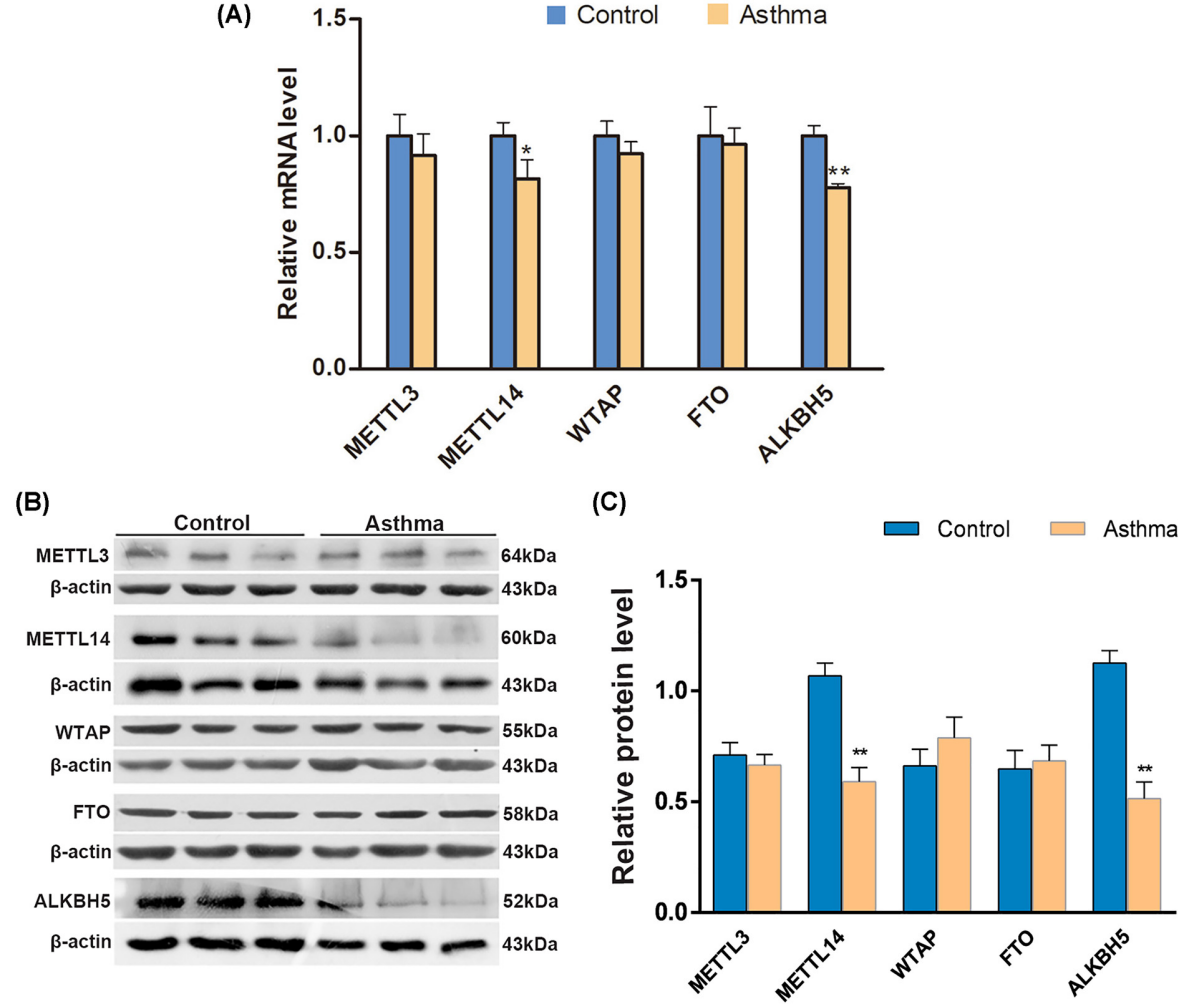
Expression of METTL3, METTL14, FTO, WTAP, ALKBH5, METTL14 (**A**) Quantitative real-time PCR for METTL3, METTL14, FTO, WTAP, ALKBH5, METTL14 level in mRNA. METTL14 (*P*=0.032) of and ALKBH5 (*P*=0.001) were down-regulated in lung of allergic asthma compared with the control group. Results are shown as fold-change of control. Data are expressed as mean ± SD. Data were analyzed using the Mann–Whitney U test. *P<0.05 or **P<0.01 versus control group (*n*=3). (**B**) Western blotting for METTL3, METTL14, FTO, WTAP, ALKBH5, METTL14 level in protein. (**C**) Optical density values for the protein expression relative to β-actin. Data are expressed as mean ± SD. **P<0.01 versus control group (*n*=6). Abbreviation: SD: standard deviation.

Five major enzymes responsible for m^6^A modification (METTL3, METTL14, WTAP, FTO, and ALKBH5) were tested for mRNA and protein levels in lung (Figure 4A–C). In the asthma group, mRNA level of METTL14, the key methyltransferase responsible for m^6^A modifications, was significantly decreased compared with the control group (*P*=0.032). ALKBH5, the major demethyltransferase, was also significantly decreased (*P*=0.001). METTL14 and ALBHK5 are also down-regulated at protein level in the group of chronic allergic asthma. While METTL3, WTAP, and the other major demethyltransferases (FTO) were not significantly dysregulated in mRNA or protein level.

### Selection and verification of target genes in m^6^A modification profiles

**Figure 5:**
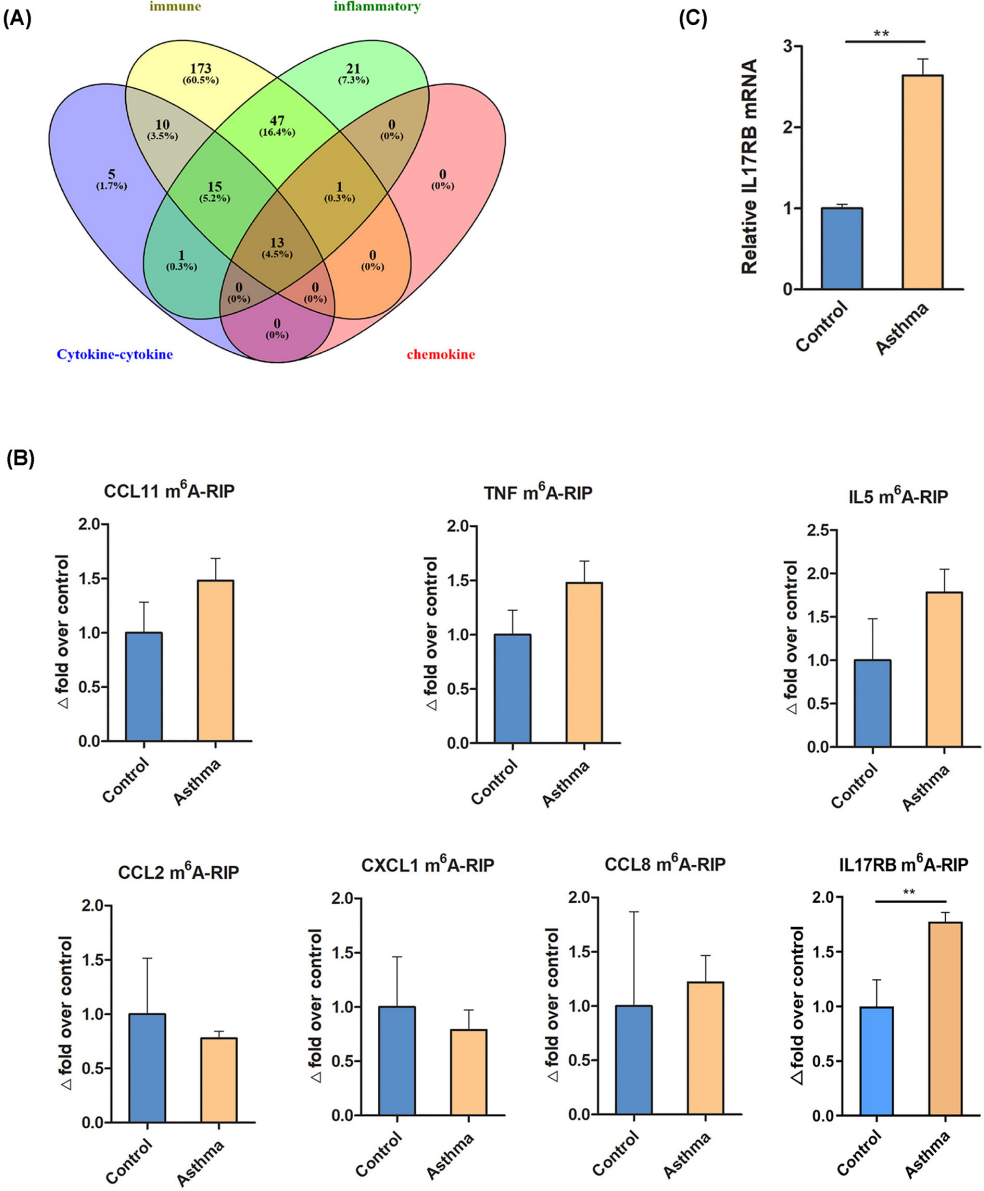
Selection and verification of target genes in m^6^A modification profiles (**A**) 39 m^6^A-hypermethylated mRNA coexpressed in interaction of cytokine–cytokine receptor interaction pathway (KEGG analysis data) and immune, inflammation, chemokine (GO analysis data), respectively. (**B**) Seven target mRNAs of m^6^A-hypermethylated were detected by quantitative real-time PCR. IL17RB mRNA was hypermethylated in lung of asthmatic group in comparion with that in control group. Data are expressed as mean ± SD. Data were analyzed using the Mann–Whitney U test. **P<0.01 versus control group (*n*=3). (**C**) IL17RB mRNA level in lung of asthmatic group was significantly increased in comparion with that in control group. Data are expressed as mean ± SD. Data were analyzed using the Mann–Whitney U test. **P<0.01 versus control group (*n*=3). Abbreviation: SD: standard deviation.

As ALKBH5 was significantly decreased (*P*=0.001), we selected the target genes in the increased m^6^A-methylated profile. Thirty-nine hypermethylated mRNAs were common to the cytokine–cytokine receptor interaction pathway (KEGG analysis data) and to immune and inflammatory chemokine-related biological processes (GO analysis data) (Figure 5A). Seven hypermethylated (CCL11, TNF, IL5, CCL2, CXCL1, CCL8, IL17RB) mRNAs (>twofold compared with control) were finally selected, as their mRNAs have been clearly reported as being associated with asthma [26–31]. We validated the seven hypermethylated genes by methylated RNA immunoprecipitation and real-time PCR. The results demonstrated that IL17RB mRNA was hypermethylated in lung of the asthmatic group in comparison with the control group (Figure 5B). Moreover, we demonstrated that IL17RB mRNA level in lung in the asthma group was also higher than that of the control group (Figure 5C).

## Discussion

Many studies have recognized that m^6^A modifications are related in various diseases, including tumor, metabolism, cardiac ischemia, stroke, lung fibrosis, high level PM2.5 exposure, and diabetes [7,9,22,32–34]. However, the role of this newly emerging layer of epigenetics in allergic asthma has not been clarified. The present study sought to evaluate the m^6^A methylation level in mRNAs and lncRNAs in lung tissue of mice with allergic asthma. We experimented with the chronic model of allergic asthma in mice, then performed transcriptome-wide m^6^A profiles by immunoprecipitated methylated RNAs with microarrays. The results showed that 1369 mRNAs and 176 lncRNAs were hypermethylated, and 197 mRNAs and 30 lncRNAs were hypomethylated (>1.5-fold compared with control) in lung of mice with allergic asthma. This suggests that chronic allergic asthma could alter m^6^A-modified transcripts, and potentially play a role in pathophysiologic processes of asthma, and find a new therapeutic target. Recent evidence demonstrates that m^6^A can induce structural changes to RNAs, thus regulating their accessibility to RBPs [35]. This also indicates that m^6^A methylation in lncRNAs may lead to their disrupted scaffolding function in allergic asthma.

GO and KEGG pathway analyses were performed to explore the potential functions of altered m^6^A-modified transcripts. KEGG pathways analysis showed that cytokine-cytokine receptor interaction, including chemokines, class I/II helical cytokines, IL17-like cytokines, TNF family, and TGF-β family exhibited increased m^6^A methylation, while it was decreased in neuroactive ligand–receptor interaction. Chemokines, IL17-like cytokines, and TNF family are known to regulate airway inflammation of asthma [26,30,31]. Interestingly, GO analysis revealed that immune response, defense response, and inflammatory response were the major biological processes modulated by the differentially m^6^A-methylated mRNAs in lung of mice with allergic asthma. Currently, drugs used for asthma therapy can relieve inflammation, but in clinical studies, none of them could inhibit airway wall remodeling [36].

The mechanism of airway wall remodeling is not well understood, yet it has been reported that the interaction between the environment and epigenetic events may play a key role in refractory asthma [37]. M^6^A modification can be modulated by cardiac function during remodeling, and by exposure to environmental toxins. This implies that m^6^A modification may contribute to immunity, inflammation, or structure remodeling in asthma. Further study is required, however, to determine the precise mechanism by which m^6^A modification affects pathophysiologic processes of allergic asthma.

M^6^A is a highly dynamic epitranscriptomic modification that regulates mRNA splicing, export, localization, translation, and stability [11,12]. Expression of METTL3, METTL14, WTAP, and FTO and ALKBH5 is associated with PM2.5 exposure and lung tumors [7,38]. We determined the level of METTL3, METTL14, WTAP, and FTO, ALKBH5 in mRNA and in protein. The m^6^A writers METTL14 and the m^6^A eraser ALKBH5 are decreased significantly both in mRNA and protein level in lung of mice with allergic asthma compared with control. These findings indicate that METTL14 and ALKBH5 may contribute to allergic asthma. Next, we anchored the increased m^6^A methylation data according to the *P*-value (ALKBH5, *P*<0.01). Of the seven target genes of m^6^A hypermethylation we tested, IL17RB mRNA was hypermethylated in lung of the asthmatic group in comparison with the control group. IL-17RB activation can regulate gene expression responsible for the inflammatory and remodeling in asthma by the JNK, MAPK, NF-κB pathways [31]. Thus, the m^6^A methylation of IL17RB mRNA, regulated by demethylase ALKBH5, may play an important role in the pathogenesis of allergic asthma, and might be a new therapeutic target. Moreover, the precise functions of METTL14, ALKBH5, and m^6^A methylation of IL17RB mRNA and lncRNAs in asthma need to be further elucidated.

## Data Availability

The author agree on sharing the present study’s data and its deposit in public repositories. See microarray data in https://www.ncbi.nlm.nih.gov/geo/query/acc.cgi?acc=GSE205471

## Abbreviations

DE: differentially expressed
GO: gene ontology
H&E: hematoxylin and eosin
lncRNA: long noncoding RNA
m6A: N6-methyladenosine
MeRIP-qRT-PCR: methylated RNA immunoprecipitation-quantitative polymerase chain reaction
OVA: ovalbumin
RBP: RNA-binding protein
Th1: helper T cell 1
Th17: helper T cell 17
Th2: helper T cell 2
Treg: regulatory T cell
